# Lessons learned from 12 years using the Woven Endobridge for the treatment of cerebral aneurysms in a multi-center series

**DOI:** 10.1038/s41598-024-75064-2

**Published:** 2024-10-16

**Authors:** Lukas Goertz, Thomas Liebig, Eberhard Siebert, David Zopfs, Lenhard Pennig, Muriel Pflaeging, Marc Schlamann, Alexandra Radomi, Franziska Dorn, Christoph Kabbasch

**Affiliations:** 1 Department of Radiology and Neuroradiology, Faculty of Medicine and University Hospital, University of Cologne, Kerpener Strasse 62, Cologne, 50937 Germany; 2 Institute of Neuroradiology, University Hospital Munich (LMU), Marchioninistrasse 15, Munich, 81377 Germany; 3 Department of Neuroradiology, University Hospital of Berlin (Charité), Charitéplatz 1, Berlin, 10118 Germany; 4 Department of Neuroradiology, University Hospital of Bonn, Venusberg-Campus 1, Bonn, 53127 Germany

**Keywords:** Aneurysm occlusion, Angiographic, Flow-disruption, Intrasaccular, Stroke, Cerebrovascular disorders, Brain imaging, Neurosurgery

## Abstract

Intrasaccular flow disruption with the Woven Endobridge (WEB) has become a well-established endovascular technique for the treatment of intracranial aneurysms. This study presents our 12-year experience with the WEB and evaluates its evolving indications, procedures, and outcomes. A consecutive series of 324 aneurysms treated with WEB between 2011 and 2023 at three neurovascular centers was retrospectively analyzed and the study group was divided into four treatment periods. Uni- and multivariate analyses were performed to evaluate factors associated with technical success, thromboembolic complications, and angiographic outcome. The mean aneurysm size was 7.0 ± 3.6 mm and decreased during the study period, while the proportion of atypical sites for WEB implantation increased. WEB implantation was technically successful in 96.0%, and the ratio of WEB width to dome width increased during the study period, indicating oversizing. The neurological complication rate was 4.9% (1.5% major, 3.4% minor) and the mid-term complete occlusion rate was 60.6% (81.9% adequate occlusion), with no statistical differences in either outcome measure between the study periods. In multivariate analyses, the use of WEB 17 was associated with increased technical success (HR: 7.4, 95%CI: 2.4-23.6, *p*<0.01), whereas ruptured aneurysm status (HR: 2.5, 95%CI: 1.0-6.0, *p*=0.04) and the use of additional stents (HR: 4.8, 95%CI: 1.6-14.4, *p*<0.01) predicted thromboembolic complications. Appropriate oversizing of the WEB favored mid-term complete occlusion (HR: 10.5, 95%CI: 1.3-83.3, *p*=0.03). The results suggest an expansion of the indications for WEB implantation and highlight the importance of oversizing for treatment efficacy.

**Supplementary Information:**

The online version contains supplementary material available at 10.1038/s41598-024-75064-2.

## Introduction

With the clinical introduction of the Woven Endobridge (WEB; Microvention, Aliso Viejo, CA, USA) in Europe in 2010, intrasaccular flow disruption has become a well-established modality for the treatment of intracranial aneurysms^1^. In 2018, the Food and Drug Administration (FDA) approved WEB for the treatment of aneurysms located at the terminus of the internal carotid artery (ICA), the bifurcation of the middle cerebral artery (MCA), the anterior communicating artery (Acom), and the tip of the basilar artery (BA)^2^. Several prospective good clinical practice studies have demonstrated a favorable safety and efficacy profile of the WEB^3–7^. Since 2010, the WEB technique has evolved, with the most important finding being that moderate oversizing of the WEB device improves angiographic outcomes^8^. In addition, the limitations and pitfalls of WEB treatment have been better understood, such as the potential occurrence of neck remnants as a result of “WEB compression” during follow-up^9^. During this time, the WEB device and delivery system have undergone several technical refinements to make the WEB more flexible and accessible to low-profile catheters. In particular, the low-profile WEB 17 system has helped expand the indications for WEB treatments, as its flexible structure and low-profile delivery system allow for the treatment of a variety of distant and sidewall aneurysms^10^.

Since 2011, we have been using the WEB device with increasing frequency and expanding its indications beyond the FDA-approved sites. The current study reviews our multicenter experience with this device and examines how the WEB technique, indications for use, and outcomes have evolved. Risk factors for treatment failure, complications, and angiographic outcomes will also be evaluated.

## Results

### Patient and aneurysm characteristics

**Figure 1:**
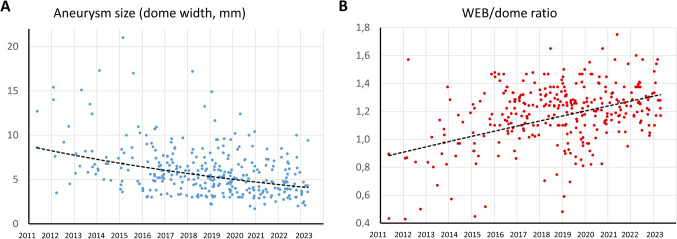
Development of aneurysm size (**A**) and WEB/dome ratio (**B**) over the study period.

During the study, 319 patients underwent WEB treatment for 324 aneurysms in 322 procedures. The mean age was 59.2 ± 11.5 years (range: 21–87) and 227 (71.2%) were female. Aneurysm details are shown in Table 1. Ruptured aneurysms were treated in 87 (27.3%) patients, of whom 24 (27.6%) had a World Federation of Neurosurgical Societies [WFNS] score of 1, 15 (17.2%) a score of 2, 21 (24.1%) a score of 3, 12 (13.8%) a score of 4, and 15 (17.2%) a score of 5. The radiologic extent of subarachnoid hemorrhage was graded with the Fisher score, with a score of 1 in 6 (6.7%) cases, a score of 2 in 11 (12.6%), a score of 3 in 30 (34.5%), and a score of 4 in 40 (46.0%). In the first quarter of the study period, 10 (3.1%) large aneurysms with a dome width > 11 mm were treated with the WEB as an individual healing attempt as part of a multimodal treatment concept. Of these, 6 (1.9%) aneurysms were partially thrombosed. Due to poor angiographic results, this treatment strategy was subsequently discontinued. Therefore, these aneurysms were removed from the subanalysis of study quarters to improve comparability. Table 2 shows the evolution of selected aneurysm, procedure, and outcome parameters over the 4 study quarters. The number of WEB treatments increased from 87 aneurysms in the first half of the study period to 237 in the second half. From the first to the last quarter, the percentage of atypical locations increased from 5.9 to 38.1% (*p* = 0.02), while the mean dome width decreased from 7.7 mm to 5.0 mm (*p* < 0.01, Fig. 1A) and the mean neck width decreased from 4.4 mm to 3.6 mm (*p* = 0.01).

**Table 1:** Baseline aneurysm characteristics.

Parameter	Value (*n* = 324)
Ruptured aneurysm status	87 (26.9%)
Partially thrombosed	6 (1.9%)
Recurrent aneurysms	27 (8.3%)
Aneurysm location	
Anterior circulation	214 (66.0%)
Anterior communicating artery	100 (30.9%)
Pericallosal artery	12 (3.7%)
Middle cerebral artery-bifurcation	40 (12.3%)
Middle cerebral artery-M1	2 (0.6%)
Internal carotid artery	
Terminus	9 (2.8%)
Paraophthalmic	20 (6.2%)
Posterior communicating	31 (9.6%)
Posterior circulation	110 (34.0%)
Basilar artery tip	83 (25.6%)
Basilar artery trunk	3 (0.9%)
Vertebral artery	1 (0.3%)
Superior cerebellar artery	7 (2.2%)
Posterior inferior cerebellar artery	16 (4.9%)
Bifurcation location	232 (71.6%)
Typical location	224 (69.1%)
Aneurysm size	
Dome width (mm)	6.0 ± 2.9 (range: 1.7–27.0)
Height (mm)	6.5 ± 3.5 (range: 1.5–27.2)
Maximum diameter (mm)	7.0 ± 3.6 (range: 2.0–27.2)
Neck width (mm)	4.0 ± 1.7 (range: 1.2–12.2)
Dome-to-neck ratio	1.5 ± 0.6 (range: 0.6–6.8)
Aspect ratio	1.7 ± 0.8 (range: 0.4-8.0)
Wide neck	308 (95.1%)

**Table 2:** Time course of selected parameters for aneurysms < 11 mm. The study period was divided into 4 time intervals. Large aneurysms were excluded for better comparability.

	Time period				*p*-value
	2011–2013	2014–2016	2017–2019	2020–2023	
Cases	17	60	124	113	
Ruptured aneurysms	4 (23.5%)	17 (28.3%)	29 (23.4%)	30 (26.5%)	0.87
Typical locations	16 (94.1%)	44 (73.1%)	94 (75.8%)	70 (61.9%)	0.02
Aneurysm dome width (mm)	7.7 ± 2.6	6.2 ± 2.1	5.6 ± 1.9	5.0 ± 2.1	< 0.01
Aneurysm neck width (mm)	4.4 ± 1.0	4.4 ± 1.7	4.0 ± 1.5	3.6 ± 1.7	0.01
Treatment failure	2 (11.8%)	6 (10.0%)	0 (0%)	5 (4.4%)	0.01
Mean WEB/dome ratio	0.8 ± 0.3	1.0 ± 0.2	1.1 ± 0.2	1.1 ± 0.2	< 0.01
Adjunctive coiling	3 (17.6%)	2 (3.3%)	4 (3.2%)	3 (2.7%)	0.01
Adjunctive stents	2 (11.8%)	12 (20.0%)	3 (2.4%)	4 (3.5%)	< 0.01
Fluoroscopy time (min)	23.1 ± 18.9	26.9 ± 16.5	27.6 ± 21.4	21.7 ± 15.6	0.13
Thromboembolic complications	2 (11.8%)	7 (11.7%)	9 (7.2%)	5 (4.4%)	0.31
Complete occlusion at mid-term follow-up	8/16 (50.0%)	34/57 (59.6%)	64/95 (67.4%)	44/80 (55.0%)	0.30
Adequate occlusion at mid-term follow-up	10/16 (62.5%)	46/57 (80.7%)	82/95 (86.3%)	62/80 (77.5%)	0.12
Retreatment	4/16 (25.0%)	6/57 (10.5%)	2/95 (2.1%)	4/80 (5.0%)	< 0.01

### Procedural details

**Figure 2:**
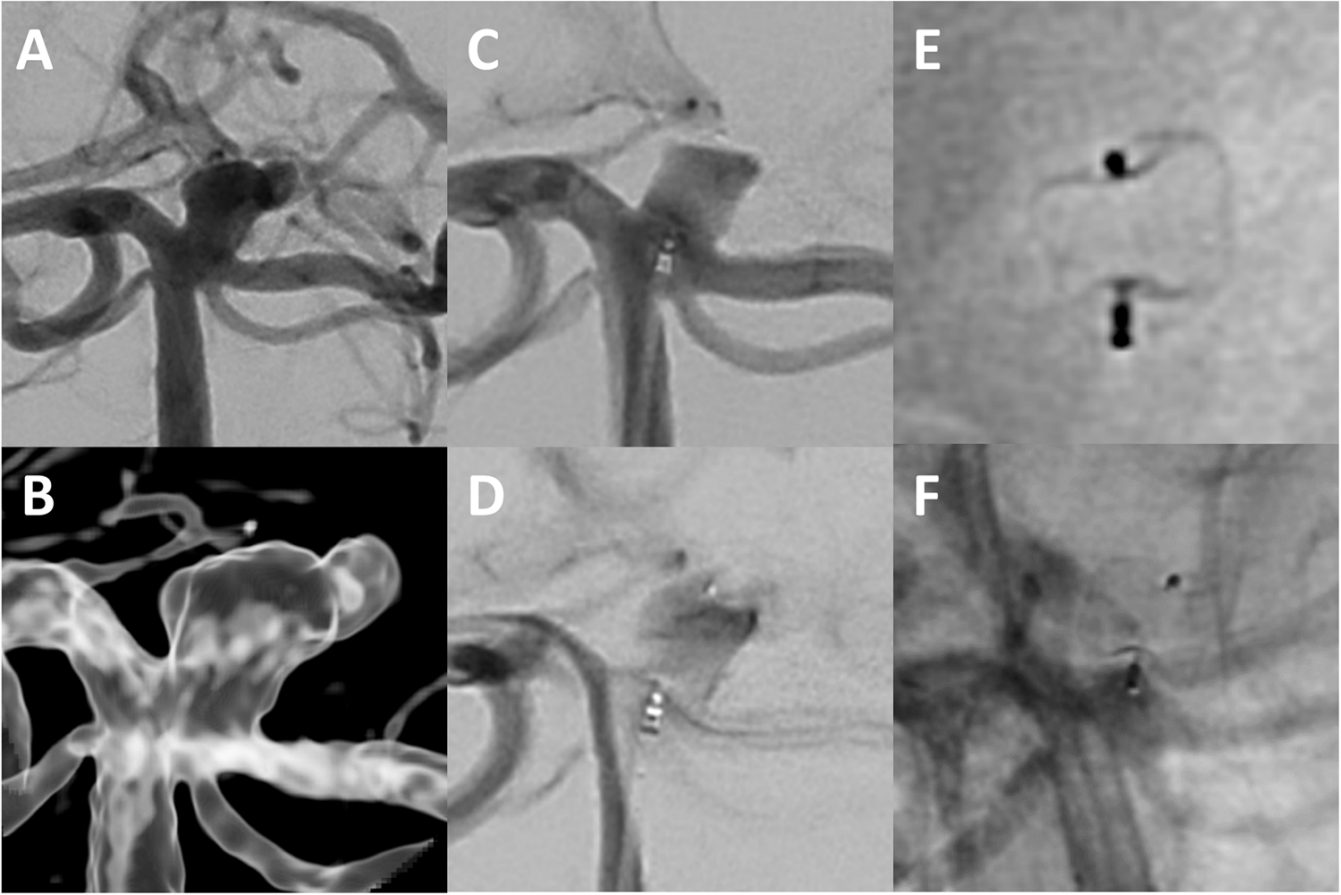
Digital subtraction angiography (**A**) and 3D reconstructions from rotational angiography (**B**) show a ruptured basilar tip aneurysm (dome width: 5.2 mm, aneurysm height: 8.5 mm, neck width: 3.4 mm) representing a “typical” WEB location. The aneurysm has an irregular shape and a rupture bleb at the top. A WEB SL 6x3 mm was deployed via a VIA 17 microcatheter (**C**), showing immediate contrast retention within the device, which is necessary to prevent aneurysm re-rupture (**D**). Enlarged unsubtracted images show how the initial cylindrical shape of the WEB changes and adapts to the shape of the aneurysm (**E**). The aneurysm neck is sealed with the base of the WEB. Nine-month angiographic follow-up shows complete occlusion of the aneurysm (**F**).

**Figure 3:**
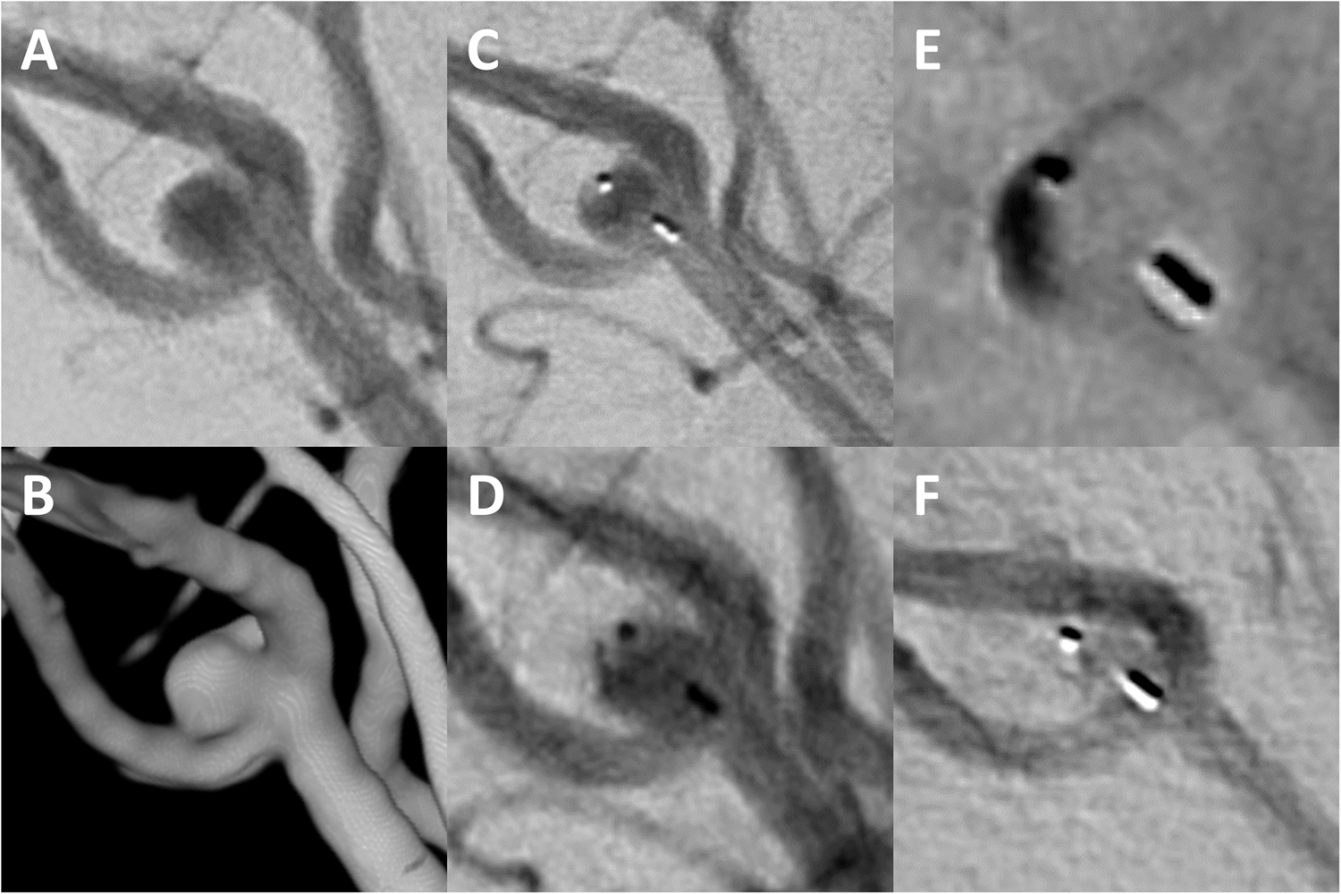
Digital subtraction angiography (**A**) and 3D reconstructions of rotational angiography (**B**) show a small unruptured aneurysm (dome width: 2.7 mm, neck width: 2.0 mm) located at the right pericallosal artery, an “atypical” site for WEB implantation. A low profile (0.017", VIA 17) microcatheter was advanced into the aneurysm and a 3.5x2 mm WEB was deployed (C). After detachment, the base of the WEB seals the aneurysm neck (**D**) and there is immediate contrast retention within the WEB (**E**). The six-month angiographic follow-up shows continued filling of the WEB (**F**). The patient was subjected to further angiographic control as there was no compression of the WEB and the aneurysm was not ruptured. In addition, the remote location, the small size of the aneurysm, the small diameter of the parent artery and the wide neck are other factors that make (re-) treatment with other endovascular modalities difficult.

The procedural characteristics are summarized in Table 3. Among 311 (96.0%) cases with successful WEB implantation, 275 (88.4%) aneurysms were treated with WEB alone, 18 (5.8%) with adjunctive coiling, and 23 (7.4%) with adjunctive stenting. Between the first and last quarter of the study period, the treatment failure rate decreased significantly from 11.8 to 4.4% (*p* = 0.01), while the WEB/dome ratio increased from 0.8 to 1.1 (*p* < 0.01, Fig. 1B). Additional coiling (17.6% vs. 2.7%, *p* = 0.01) and stenting (11.0% vs. 3.5%, *p* < 0.01) were performed significantly more often in the first quarter than in the last quarter (Table 2). Fluoroscopy time remained constant throughout the study period (*p* = 0.13). In univariate analysis, the use of the WEB 17 system was associated with treatment success (HR: 7.4, 95%CI: 2.4–23.6, *p* < 0.01). Figures 2 and 3 show cases of successful WEB embolization of aneurysms in a typical and atypical location, respectively.

**Table 3:** Procedural characteristics.

Parameter	Value (*n* = 324)
Treatment success	311 (96.0%)
WEB alone	275/311 (88.4%)
Adjunctive coiling	18/311 (5.8%)
Adjunctive stents	23/311 (7.4%)
WEB type	
Double-layer (DL)	16 (4.9%)
Single-layer (SL)	243 (75.0%)
Single-layer sphere (SLS)	65 (20.1%)
Delivery system	
0.017” (WEB 17)	188 (58.0%)
0.021” (WEB 21)	63 (19.4%)
0.027” (WEB 27)	37 (11.4%)
0.033” (WEB 33)	36 (11.1%)
WEB width (mm)	6.0 ± 1.9 (range: 3.0–11.0)
WEB/dome ratio	1.1 ± 0.2 (range: 0.7–1.7)
Fluoroscopy time (min)	26.1 ± 19.3 (range: 2.4–97.4)

### Complications and clinical outcome

The overall procedural complication rate was 9.9%, with major neurological complications in 1.5% and minor neurological complications in 3.4%. Thromboembolic events occurred in 25 cases (7.7%), resulting in cerebral infarction in 9 (2.8%), of which 6 (1.9%) were symptomatic. Hemorrhagic complications occurred in 5 patients (1.5%). In the subanalysis, the rate of thromboembolic events was higher in the first half of the study (9/77, 11.7%) than in the second half (14/237, 5.9%), but this difference was not statistically significant (*p* = 0.09). No other complications such as delayed aneurysm rupture or ischemic stroke were observed during clinical follow-up. In univariate analysis, the following variables were associated with procedural thromboembolic complications: aneurysm rupture status (HR: 2.5, 95%CI: 1.1-5. 7, *p* = 0.028), WEB 21 system (HR: 2.5, 95%CI: 1.1–6.1, *p* = 0.03), WEB 33 system (HR: 2.8, 95%CI: 1.1–7.6, *p* = 0.03), and additional stents (HR: 6.6, 95%CI: 2.4–18.0, *p* < 0.01). In multivariate analysis, ruptured aneurysm status (HR: 2.5, 95%CI: 1.0–6.0, *p* = 0.04) and additional stents (HR: 4.8, 95%CI: 1.6–14.4, *p* < 0.01) were independently associated with thromboembolic complications.

Clinical outcome was determined on a per-procedure basis (Table 4). A favorable outcome was achieved in 98.3% (231/235) of elective cases and 55.2% (48/87) of ruptured cases. Overall mortality was 0.4% (1/235) for unruptured aneurysms and 5.7% (5/87) for ruptured aneurysms. The death in the unruptured group occurred in the early study period and consisted of an iatrogenic aneurysm perforation with a WEB SL and subsequent brain death.

**Table 4:** Clinical outcome defined by the modified Rankin scale score at discharge.

modified Rankin scale score	Unruptured (*n* = 235)	Ruptured (*n* = 87)
0	218 (92.8%)	33 (37.9%)
1	8 (3.4%)	10 (11.5%)
2	5 (2.1%)	5 (5.7%)
3	2 (0.9%)	5 (5.7%)
4	0 (0%)	11 (12.6%)
5	1 (0.4%)	18 (20.7%)
6	1 (0.4%)	5 (5.7%)

### Angiographic outcome

**Figure 4:**
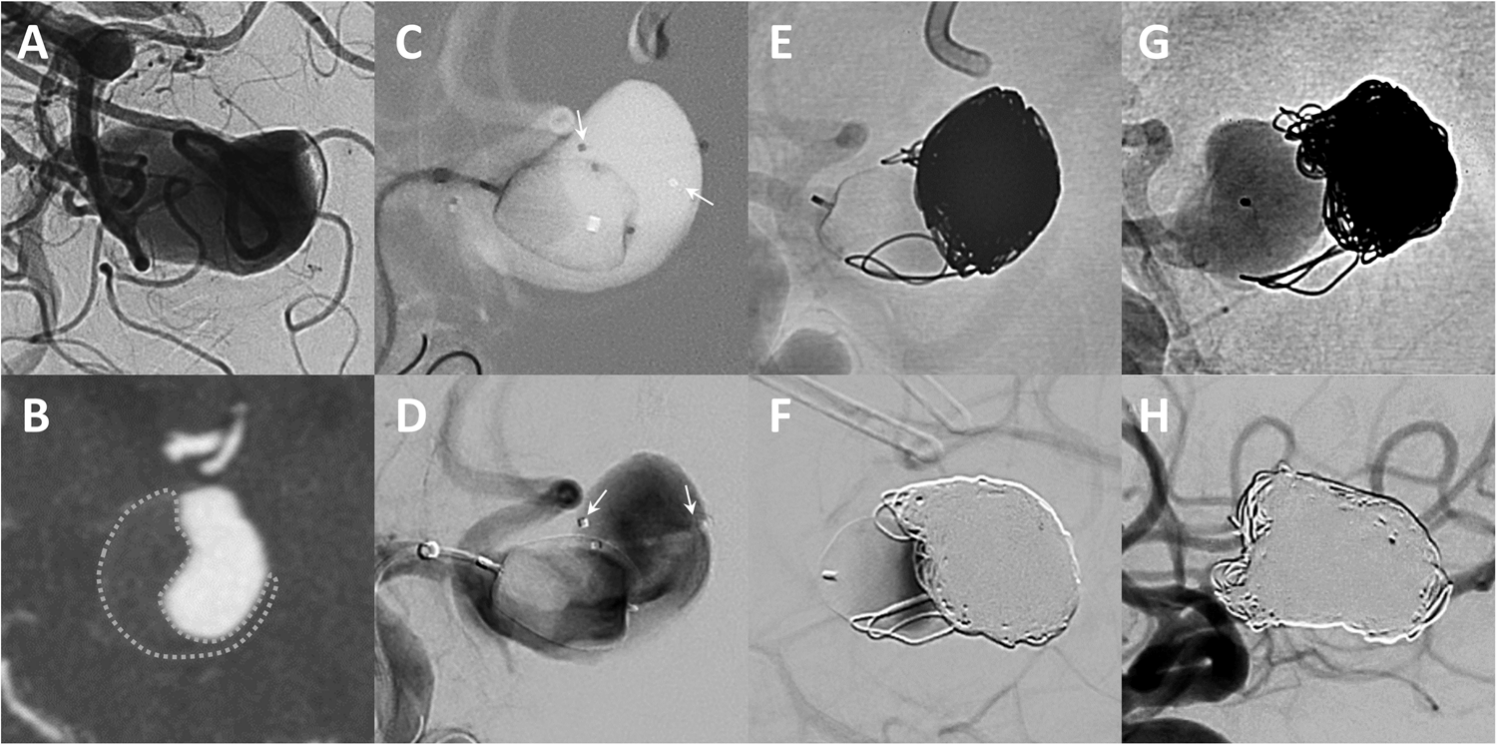
Digital subtraction angiography shows a giant ruptured aneurysm (dome width: 14 mm, height: 25 mm, neck width: 7.5 mm) of the anterior communicating artery (**A**). The aneurysm is partially thrombosed as shown on computed tomography angiography (**B**), the outline of the thrombus is highlighted with a dotted line in this image. As the aneurysm was ruptured, stent-assisted coiling was not chosen to avoid antiplatelet therapy. Instead, WEB-assisted coiling was chosen with the intention of placing the WEB at the base of the aneurysm to seal the aneurysm neck. With this intention, a WEB SLS 11 mm was deployed via a 0.033" microcatheter at the aneurysm base, and a second microcatheter (arrows) was introduced into the aneurysm sac using the Jailing technique for subsequent coil embolization (**C**: roadmap, **D**: subtracted images). The aneurysm dome was then embolized with 17 coils, resulting in an almost complete occlusion (**E**). Immediate control angiography shows incipient contrast retention within the WEB (**F**). However, 6-month follow-up angiography shows migration of the coils and WEB within the pre-existing aneurysm thrombus and both coil and WEB compaction resulting in extensive aneurysm recanalisation with a large aneurysm remnant (**G**). The aneurysm was then retreated with stent-assisted coiling to achieve complete occlusion (**H**).

Angiographic results are reported on an as-treated basis, with aneurysms retreated after the mid-term follow-up included in the long-term follow-up as aneurysm remnants (Table 5). The complete and adequate occlusion rates were 60.6% and 81.9%, respectively, at mid-term (mean follow-up 6 months) and 56.1% and 72.4%, respectively, at long-term (mean follow-up 21 months). Of the 117 aneurysms available for mid-term and long-term follow-up, progressive aneurysm occlusion was observed in 12 (10.3%) cases and recanalization in 12 (10.3%) cases. Of the recanalizations, six were neck remnants and six were aneurysm remnants. The retreatment rates were 7.1% (18/249) at mid-term follow-up and 1.6% (2/123) at long-term follow-up. Three large aneurysms were treated twice. Retreatment strategies included stent-assisted coiling in 9 cases, flow diverter implantation in 7, coiling in 5, stent implantation in 1, and WEB plus coiling in 1. Mid-term adequate occlusion rates did not differ significantly between study quarters (*p* = 0.12). However, retreatment rates decreased from 13.7% (10/73) in the first half of the study to 3.4% (6/175) in the second half (*p* < 0.01). The following variables were associated with aneurysm remnants at mid-term follow-up: partially thrombosed aneurysms (HR: 2.5, 95%CI: 2.2–2.9, *p* < 0.01), large aneurysms (HR: 12.2, 95%CI: 1.5–100.0, *p* < 0.01), recurrent aneurysms (HR: 2.8, 95%CI: 1.1–6.8, *p* = 0.02), WEB 33 system (HR: 2.7, 95%CI: 1. 2-6.1, *p* = 0.01), additional coiling (HR: 4.3, 95%CI: 11.4–13.2, *p* = 0.01), greater dome width (HR: 1.2, 95%CI: 1.1–1.3, *p* < 0.01), greater height (HR: 1. 1, 95%CI: 1.0-1.2, *p* < 0.01), greater neck width (HR: 1.3, 95%CI: 1.1–1.5, *p* < 0.01), and smaller WEB/dome ratio (HR: 7.2, 95%CI: 1.4–37.0, *p* = 0.017). Due to high collinearity between the factors, large aneurysm size, recurrent aneurysms, WEB 33 system, adjunctive coiling, and WEB/dome ratio were selected for inclusion in the multivariate analysis: A higher WEB/dome ratio was the only independent factor independently associated with adequate aneurysm occlusion (HR: 10.5, 95%CI: 1.3–83.3, *p* = 0.03). The following variables were associated with aneurysm recurrence between mid- and long-term follow-up: ruptured aneurysms (HR: 4.7, 95%CI: 1.3–16.6, *p* = 0.01), partially thrombosed aneurysms (HR: 5.5, 95%CI: 1.0-33.5, *p* = 0.04), and larger aspect ratio (HR: 1.7, 95%CI: 1.1–2.9, *p* = 0.03). On multivariate analysis, ruptured aneurysm status remained an independent factor associated with aneurysm recurrence (HR: 3.8, 95%CI: 1.1–14.3, *p* = 0.04). Figure 4 shows an illustrative case of WEB embolization of a giant, partially thrombosed aneurysm that recurred and had to be retreated.

**Table 5:** Angiographic outcome at mid- and long-term follow-up (FU).

	Mid-term FU	Long-term FU
Mean FU period (months)	5.7 ± 3.1	21.4 ± 8.0
Occlusion status	*N* = 249	*N* = 123
Complete occlusion	151 (60.6%)	69 (56.1%)
Neck remnant	53 (21.3%)	20 (16.3%)
Aneurysm remnant	45 (18.1%)	34 (27.6%)
Retreatment	18 (7.1%)	2 (1.6%)
Progression between mid- and long-term FU		*N* = 117
Stable occlusion	-	93 (79.5%)
Progressive occlusion	-	12 (10.3%)
Recurrence	-	12 (10.3%)

## Discussion

This 12-year study highlights the evolving nature of WEB treatment and may serve as a real-world benchmark for treatment outcomes. Over time, off-label use for atypical locations increased and treatment of smaller aneurysms became more common. Technical failures decreased and fewer aneurysms required additional stents or coils. In particular, the WEB/dome ratio increased significantly, indicating a progressive use of oversizing. Mid-term complete and adequate occlusion rates were 59% and 79% for the entire cohort, respectively, without significant differences between the study quarters. Clinical complications occurred in 4.9% with a major complication rate of 1.5%. These results are consistent with the WorldWideWEB Consortium study, which recruited patients over a similar time period^1^.

Increasing experience and technical refinement have allowed the WEB to be used in locations beyond the FDA-approved bifurcation locations. The introduction of VIA 17 microcatheters with 45° and 90° angled tips and the ability to shape the tip facilitates catheterization of aneurysms with angles up to 90°, such as distal and sidewall aneurysms^11^. The WEB 17 is available in a small 3 × 2 mm device size, allowing treatment of aneurysms as small as 2.5 × 2.0 mm^4,12^. As a result, FDA-approved locations decreased from 94% (first quarter) to 62% (last quarter) in this study, and average aneurysm size decreased from 7.7 mm to 5.0 mm. Outcome and complication rates showed no statistical difference between typical and atypical locations, consistent with previous studies^13–16^.

Initially, the manufacturer’s guidelines recommended WEB treatment for aneurysms up to 11 mm because the largest available WEB width is 11 mm. However, considering the oversizing of the device, aneurysm widths of 2 to 10 mm may be more appropriate for WEB treatment^8^. The WEB size can be determined using the manufacturer’s sizing chart, which allows for a moderate oversize of 1–2 mm of the device width. This allows lateral compression for complete aneurysm wall contact. Residual contrast filling of the dome at immediate angiographic follow-up is not necessary due to progressive thrombosis. Consistent with previous findings, multivariate analyses showed that WEB oversizing was independently correlated with improved mid-term occlusion results without additional morbidity^10,17^.

Even with consistent WEB oversizing, a mechanism called “WEB compression” can result in neck remnants, which occur in approximately 30% of cases approximately 6 months after treatment^18^. The WorldWideWEB Consortium has noted a decrease in the rate of WEB compression over time, likely due to more consistent oversizing and technological advances in the device and delivery system^19^. In the Pierot et al. study, only 11% of neck remnants progressed to aneurysm remnants within 1–2 years of follow-up^20,21^. Similarly, in the present study, worsening of aneurysm occlusion was observed in 10.2% beyond mid-term follow-up, with ruptured aneurysm status being an independent risk factor. Typically, stable neck remnants after WEB implantation do not require retreatment, leading some authors to classify complete occlusion and neck remnants as “adequate” occlusion^18,22,23^. In addition, specific scales such as WOS (WEB Occlusion Scale) and BOSS (Bicêtre Occlusion Scale) further differentiate aneurysm remnants^24^.

Our study showed a cumulative retreatment rate of 15% compared to 12% in the two WEBCAST studies^21^. Interestingly, there was a marked decrease in retreatment from 13.7% (first half) to 3.4% (second half), possibly related to a slight, non-significant increase in adequate occlusion rates and a growing awareness that neck remnants may not require retreatment. Various retreatment strategies have been described in the literature, including stent-assisted coiling, stand-alone stenting, flow diversion, and WEB treatment with or without stenting and clipping^25–27^. While retreatment is generally safe and effective, it is important to recognize that methods such as clipping and stent-assisted procedures compromise the advantages of WEB as a minimally invasive, intrasaccular approach.

Although acetylsalicylic acid (ASA) is not mandatory during WEB treatment, elective cases received ASA 100 mg for 5–7 days prior to the procedure to facilitate additional stent implantation or conversion to stent-assisted coiling if needed. Pretreatment with ASA may reduce thromboembolic events, although clinical data do not yet support this notion.

The WEB device is typically used as a stand-alone treatment for intracranial aneurysms, but there are instances where additional devices may be beneficial when used in combination with the WEB device. An additional stent may be used to maintain branch patency in the event of WEB protrusion. It may also be helpful in aneurysms where a branch arises from the neck of the aneurysm and there is a possibility of the WEB device protruding into it. In these cases, a microcatheter is placed in the branch before the WEB is deployed, followed by deployment of the WEB device itself.

Additional coils are usually not necessary, even in lobulated aneurysms, as portions of the aneurysm not covered by the WEB will thrombose over time as long as the WEB is properly placed on the aneurysm neck. Coils may be used in selected cases of ruptured aneurysms to provide immediate occlusion of the rupture site or in cases where an aneurysm remnant is observed after WEB detachment due to inadequate oversizing. Typically, the flow disrupting effect of the WEB is sufficient to prevent early re-bleeding of ruptured aneurysms as documented in the literature. Early rebleeding rates were 0% in the CLARYS study, 1.2% in the literature review by Crinnion et al., and 2.5% in the meta-analysis by Harker et al.^28–30^. Essibayi et al. reported a late bleeding rate of 1.1%^31^. These rebleeding rates are within the range of the International Subarachnoid Aneurysm Trial (ISAT), where the 1-year rebleeding rate was 2.5% after coiling and 1.3% after surgery^32^.

During the first quarter of the study period, clinical experience with the WEB was limited and the WEB was used off-label for the treatment of large and partially thrombosed aneurysms. Two concepts were pursued in the hope of achieving more durable aneurysm occlusion: (1) filling the aneurysm sac with multiple WEBs; (2) sealing the aneurysm neck with a WEB after coiling the aneurysm sac. However, early results indicated that WEB treatment of these aneurysms resulted in poor outcomes similar to other endovascular treatment methods, as shown in Fig. 4. Therefore, we no longer use the WEB for large and partially thrombosed aneurysms^33^.

In this study, the overall thromboembolic event rate was 7.7% and decreased from 11.8% in the first quarter to 4.4% in the last quarter. There is evidence that the low-profile WEB 17 System reduces the risk of thromboembolic events^34,35^. These rates are comparable to conventional coiling and appear to be superior to stent-assisted coiling or clipping ^36–39^. Common techniques for managing thromboembolic events during WEB implantation include intra-arterial antiplatelet therapy and thrombectomy using aspiration or stent retrieval techniques.

The simplicity of WEB deployment, the safety and efficacy profile, and the lack of need for antiplatelet therapy have made WEB the preferred treatment option for unruptured aneurysms with saccular, WEB-suitable morphology at the authors’ institution. In particular, balloon- or stent-assisted coiling has been reserved for complex cases not suitable for WEB, such as irregular, lobulated aneurysms or aneurysms larger than the maximum diameter on the sizing chart (i.e., 10 mm).

### Limitations

Several limitations must be acknowledged. The study period was 12 years, during which WEB technology and experience evolved, resulting in an inhomogeneous study sample. The aneurysms were treated in only three centers with comparable treatment regimens, but the results may not be generalizable to different treatment regimens and health care systems. The retrospective design and moderate number of patients included are other limitations that reduce the generalizability of the results. Long-term angiographic follow-up was limited and not available in all cases. Angiographic evaluation was not performed by a core laboratory, which may have influenced the anatomic results.

## Conclusions

The study shows how the use of WEB devices has shifted to smaller aneurysms and broader indications over the past 12 years. The oversizing strategy has become the standard of practice for WEB deployment, and multivariate analysis demonstrated the importance of this technique in improving angiographic outcomes. Supported by comparative studies, the WEB is increasingly being used as an alternative treatment option for aneurysms amenable to coiling, stent-assisted coiling, or microsurgical clipping. Further studies will determine the long-term safety and efficacy of WEB treatment and help expand its indications.

## Methods

This is a retrospective review of consecutive patients treated with the WEB at three tertiary care centers between 2011 and 2023. The retrospective data collection was approved by the local ethics committee. Separate approval of this study was not required due to the retrospective design in accordance with institutional policy. Informed consent was also waived by the local ethics committees. The study was conducted according to the STROBE guidelines in compliance with national legislation and the World Medical Association’s Code of Ethical Principles for Medical Research Involving Human Subjects (Declaration of Helsinki).

### WEB treatment

The WEB procedure was performed using a transfemoral approach in a biplane angiosuite (Philips, Best, The Netherlands and Siemens, Erlangen, Germany). The dedicated VIA microcatheters (Microvention, Aliso Viejo, CA, USA) were used to deliver the WEB in most cases. WEB 17 and WEB 21 used 0.017” and 0.021” microcatheters for sizes ≤ 7 mm, while 8–11 mm WEBs required 0.027” or 0.033” microcatheters. Beginning in September 2020, all aneurysms ≤ 7 mm were treated with WEB 17. This study included WEB single layer (SL), double layer (DL) and single layer spherical (SLS) types. Since 2014, implant sizes were consistently chosen to be slightly larger than the aneurysm dome diameter and slightly smaller than the aneurysm height according to the sizing chart. Stents were used to assist in unfavorable anatomy, and additional coiling was used to achieve complete occlusion in atypical or large (> 11 mm) aneurysms at the discretion of the neurointerventionalist.

### Anti-aggregation therapy

In elective cases, acetylsalicylic acid (ASA) 100 mg/day was administered for ≥ 4 weeks starting 5–7 days before the procedure. A 5000 IU heparin bolus was administered after inguinal puncture, followed by 1000 IU/hour during the procedure. If stents were used, ASA 100 mg and clopidogrel 75 mg were given for ≥ 4 months, then ASA was continued. For acutely ruptured aneurysms treated with stent-assisted WEB, tirofiban infusion was used for 16–24 h, followed by loading doses of ASA (250 mg) and clopidogrel (300 mg). Maintenance antiplatelet therapy mirrored elective cases.

### Data collection

Charts were reviewed to collect patient demographics, procedural details, and procedural complications. Treatment failure was defined as inability to adequately place the WEB within the aneurysm sac with or without stent support and eventual removal, requiring treatment of the aneurysm with another endovascular modality or clipping. Procedure-related thromboembolic and hemorrhagic complications were recorded, both clinically relevant (symptomatic) complications and technical complications without clinical sequelae. Neurological complications were considered major if symptoms lasted ≥ 7 days and minor if resolved within 7 days. Clinical outcome was assessed using the modified Rankin Scale.

### Angiographic evaluation

Neurointerventionalists at each center performed angiographic assessments. Conventional four-vessel digital subtraction angiography (DSA) determined aneurysm dome width (D), height (H), neck width (N), dome to neck ratio (D/N), and aspect ratio (H/N). A wide neck was defined as *N* ≥ 4 mm and/or D/*N* ≤ 2. Large aneurysms were defined as greater than 11 mm in diameter, which is an off-label use of WEB. The WEB/dome ratio is the ratio of the WEB width to the aneurysm dome width. The anterior communicating artery, internal carotid artery terminus, middle cerebral artery bifurcation, and basilar tip were defined as “typical” locations. Aneurysm occlusion was categorized as “complete”, “neck remnant”, or “aneurysm remnant”. Complete occlusion and neck remnants were considered adequate occlusion. Mid-term follow-up was defined as 1 to 12 months, and long-term follow-up was defined as more than 12 months.

### Statistical analysis

Qualitative variables were expressed as numbers and percentages and compared using chi-squared and Fisher exact tests. Quantitative variables were presented as means with standard deviation and compared using Student’s t-test for normally distributed data or Wilcoxon-Mann-Whitney test for non-normally distributed data. The Shapiro-Wilk test was used to test for normality. In the subanalysis, the study period was divided into 4 quarters based on the date of treatment (2011–2013, 2014–2016, 2017–2019, 2020–2023). In the subanalysis, variables were compared across study quarters using one-way analysis of variance (ANOVA) for normally distributed data, Kruskal-Wallis test for non-normally distributed data, and 4 × 2 chi-squared test for qualitative variables. A p-value < 0.05 indicates a significant difference between study quarters; additional post-hoc analyses were not performed.

In addition, univariate analyses were performed to identify factors associated with treatment failure, procedural thromboembolic events, aneurysm remnants at mid-term, and recanalization between mid-term and long-term follow-up. The following parameters were included as covariates: Patient age, sex, ruptured aneurysm status, recurrent aneurysm status, partial intrasaccular thrombosis, large aneurysm size, anterior/posterior circulation location, typical/atypical aneurysm location, bifurcation/sidewall location, dome width, height, neck width, D/N ratio, aspect ratio, adjunctive coiling and/or stenting, WEB type (DL, SL, SLS), delivery system (WEB 17, 21, 27, and 33), and WEB/dome ratio. Significant factors in the univariate analysis (*p* < 0.05) were then entered into a stepwise bivariate logistic regression model to identify independent variables associated with each outcome measure. SPSS software (IBM SPSS Statistics for Windows, version 25.0, Armonk, NY, USA) was used for analysis, with *p* < 0.05 considered statistically significant.

## Supplementary Information

Supplementary Material 1.

## Data Availability

All data will be made available upon request in an anonymized manner from the corresponding author LG (email: Lukas.goertz@uk-koeln.de).

## References

[1] Dmytriw, A. A. et al. International study of intracranial aneurysm treatment using Woven EndoBridge: results of the WorldWideWEB Consortium. *Stroke*. **53**, e47–e49 (2022). 34915737 10.1161/STROKEAHA.121.037609 PMC8792251

[2] Al Saiegh, F. et al. Treatment of Acutely Ruptured Cerebral Aneurysms With the Woven EndoBridge Device: Experience Post-FDA Approval. *Neurosurgery*. **87.1**, E16-E22 (2020). 10.1093/neuros/nyaa092 PMC8929032 32357228

[3] Arthur, A. S. et al. The safety and effectiveness of the Woven EndoBridge (WEB) system for the treatment of wide-necked bifurcation aneurysms: final 12-month results of the pivotal WEB intrasaccular therapy (WEB-IT) study. *J. Neurointerventional Surg. ***11**, 924–930 (2019). 10.1136/neurintsurg-2019-014815 PMC6824604 30992395

[4] Goertz, L. et al. Low-Profile Intra-aneurysmal Flow Disruptor WEB 17 versus WEB Predecessor systems for treatment of small intracranial aneurysms: comparative analysis of Procedural Safety and Feasibility. *Am. J. Neuroradiol. ***40**, 1766–1772 (2019). 31488499 10.3174/ajnr.A6183 PMC7028555

[5] Pennig, L. et al. The Woven EndoBridge (WEB) versus Conventional Coiling for Treatment of Patients with Aneurysmal Subarachnoid Haemorrhage: Propensity Score-Matched Analysis of Clinical and Angiographic Outcome Data. *World Neurosurg*. **146**, e1326-e1334 (2020). 10.1016/j.wneu.2020.11.158 33290897

[6] Pierot, L. et al. Safety and efficacy of aneurysm treatment with WEB: results of the WEBCAST study. *J. Neurosurg. ***124**, 1250–1256 (2016). 26381253 10.3171/2015.2.JNS142634

[7] Pierot, L. et al. Safety and efficacy of aneurysm treatment with the WEB: results of the WEBCAST 2 study. *Am J Neuroradiol*. **38.6**, 1151-1155 (2017). 10.3174/ajnr.A5178 PMC7960101 28450432

[8] Goyal, N. et al. How to WEB: a practical review of methodology for the use of the Woven EndoBridge. *J. Neurointerventional Surg. ***12**, 512–520 (2020). 10.1136/neurintsurg-2019-015506 PMC7231463 32005760

[9] Herbreteau, D. et al. Are anatomic results influenced by WEB shape modification? Analysis in a prospective, single-Center Series of 39 patients with aneurysms treated with the WEB. *AJNR Am. J. Neuroradiol. ***37**, 2280–2286 (2016). 27538903 10.3174/ajnr.A4918 PMC7963884

[10] Sabuzi, F. et al. How a decade of aneurysms embolization with the Woven EndoBridge has changed our understanding and practices? *J Neuroradiol*. **50.5**, 518-522 (2023). 10.1016/j.neurad.2023.02.006 36868371

[11] Pierot, L. Ten years of clinical evaluation of the Woven EndoBridge: a safe and effective treatment for wide-Neck bifurcation aneurysms. *Neurointervention*. **16**, 211–221 (2021). 34674453 10.5469/neuroint.2021.00395 PMC8561039

[12] Pagano, P. & Cortese, J. Aneurysm Treatment with Woven EndoBridge-17: angiographic and clinical results at 12 months from a Retrospective. *2-Center Ser. ***44**, 467–473 (2023). 10.3174/ajnr.A7830 PMC10084902 36997284

[13] Aguiar, G., Caroff, J. & Mihalea, C. WEB device for treatment of posterior communicating artery aneurysms. *J. Neurointerventional Surg*. **14**, 362–365 (2022). 10.1136/neurintsurg-2021-017405 33975921

[14] Goertz, L. & Liebig, T. Treatment of proximal posterior inferior cerebellar artery aneurysms by Intrasaccular Flow disruption: a Multicenter experience. *AJNR Am. J. Neuroradiol.* **43**, 1158–1163 (2022). 10.3174/ajnr.A7566 PMC9575426 35863779

[15] Goertz, L. et al. Extending the indication of Woven EndoBridge (WEB) embolization to internal carotid artery aneurysms: a multicenter safety and feasibility study. *World Neurosurg*. **126**, e965-e974 (2019). 10.1016/j.wneu.2019.02.198 30876989

[16] Rodriguez-Calienes, A. & Vivanco-Suarez, J. Use of the Woven EndoBridge Device for Sidewall Aneurysms: Systematic Review and Meta-analysis. *AJNR Am. J. Neuroradiol.* **44**, 165–170 (2023). 10.3174/ajnr.A7766 PMC9891330 36635056

[17] Caroff, J. et al. Woven EndoBridge device shape modification can be mitigated with an appropriate oversizing strategy: a VasoCT based study. *J. NeuroInterv Surg* **14**, (2022). 10.1136/neurintsurg-2020-017232 33727411

[18] Herbreteau, D. et al. Are anatomic results influenced by WEB shape modification? Analysis in a prospective, single-Center Series of 39 patients with aneurysms treated with the WEB. *Am. J. Neuroradiol. ***37**, 2280–2286 (2016). 27538903 10.3174/ajnr.A4918 PMC7963884

[19] Dmytriw, A. A. et al. The Woven EndoBridge (WEB) Device for the Treatment of Intracranial Aneurysms: Ten Years of Lessons Learned and Adjustments in Practice from the WorldWideWEB Consortium. *Trans Stroke Res*. **14.4**, 455-464 (2022). 10.1007/s12975-022-01072-x 36066701

[20] Pierot, L. et al. Aneurysm treatment with Woven Endobridge in the cumulative population of three prospective, multicenter series: 2-year follow-up. *Neurosurgery*. **87.2**, 357-367 (2020). 10.1093/neuros/nyz557 PMC7534535 31960052

[21] Pierot, L. et al. Aneurysm treatment with the Woven EndoBridge (WEB) device in the combined population of two prospective, multicenter series: 5-year follow-up. *J. NeuroInterventional Surg. ***15**, 552–557 (2023). 10.1136/neurintsurg-2021-018414 PMC10314010 35803731

[22] Cognard, C. & Januel, A. C. Remnants and recurrences after the use of the WEB intrasaccular device in large-neck bifurcation aneurysms. *Neurosurgery*. **76**, 522–530 (2015). 25710103 10.1227/NEU.0000000000000669

[23] Sivan-Hoffmann, R. et al. One-year Angiographic Follow-Up after WEB-SL Endovascular treatment of wide-Neck Bifurcation Intracranial aneurysms. *AJNR Am. J. Neuroradiol. ***36**, 2320–2324 (2015). 26294645 10.3174/ajnr.A4457 PMC7964263

[24] Caroff, J. et al. Occlusion assessment of intracranial aneurysms treated with the WEB device. *Neuroradiology*. **58**, 887–891 (2016). 27312475 10.1007/s00234-016-1715-9

[25] Caroff, J. et al. Management of aneurysmal recurrence after Woven EndoBridge (WEB) treatment. *J NeuroInterv Surg*. **15.10**, 939-942 (2022) 10.1136/jnis-2022-019645 36288976

[26] Kabbasch, C. et al. Treatment strategies for recurrent and residual aneurysms after Woven Endobridge implantation. *J. NeuroInterventional Surg. ***11**, 390–395 (2019). 10.1136/neurintsurg-2018-014230 30154251

[27] Kranawetter, B. et al. Microsurgical clipping as a retreatment strategy for previously ruptured aneurysms treated with the Woven EndoBridge (WEB) device: a mono-institutional case series. *Acta Neurochir.* **165.7**, 1881-1889 (2023). 10.1007/s00701-023-05596-5 PMC10319653 37178247

[28] Crinnion, W. et al. The Woven Endobridge as a treatment for acutely ruptured aneurysms: a review of the literature. *Interventional Neuroradiol. ***27**, 602–608 (2021). 10.1177/1591019921991397 PMC8493343 33509013

[29] Harker, P. et al. The Woven EndoBridge device for ruptured intracranial aneurysms: international multicenter experience and updated meta-analysis. *Neuroradiology*. **63**, 1891–1899 (2021). 34031704 10.1007/s00234-021-02727-6

[30] Spelle, L. et al. CLinical Assessment of WEB device in ruptured aneurYSms (CLARYS): results of 1-month and 1-year assessment of rebleeding protection and clinical safety in a multicenter study. *J. NeuroInterventional Surg. ***14**, 807–814 (2022). 10.1136/neurintsurg-2021-017416 PMC9304095 34493578

[31] Essibayi, M., Lanzino, G. & Brinjikji, W. Safety and efficacy of the Woven EndoBridge device for treatment of ruptured intracranial aneurysms: a systematic review and meta-analysis. *Am. J. Neuroradiol. ***42**, 1627–1632 (2021). 34117016 10.3174/ajnr.A7174 PMC8423055

[32] Molyneux, A. & Group, I. S. A. T. C. International Subarachnoid Aneurysm Trial (ISAT) of neurosurgical clipping versus endovascular coiling in 2143 patients with ruptured intracranial aneurysms: a randomised trial. *Lancet*. **360**, 1267–1274 (2002). 12414200 10.1016/s0140-6736(02)11314-6

[33] Kabbasch, C., Mpotsaris, A., Reiner, M. & Liebig, T. WEB as part of a multimodality treatment in complex, large, and partially thrombosed intracranial aneurysms: a single-center observational study of technical success, safety, and recurrence. *J. Neurointerventional Surg. ***8**, 1235–1239 (2016). 10.1136/neurintsurg-2015-012126 26801945

[34] König, I. et al. *Treatment of Ruptured and Unruptured Intracranial Aneurysms with WEB 17 Versus WEB 21 Systems: Comparison of Indications and Early Angiographic Outcomes* (2020). 10.1007/s00062-020-00946-7 32880656

[35] Zhang, S. M., Liu, L. X., Ren, P. W., Xie, X. D. & Miao, J. Effectiveness, Safety and Risk factors of Woven EndoBridge device in the treatment of wide-Neck Intracranial aneurysms: systematic review and Meta-analysis. *World Neurosurg. ***136**, e1–e23 (2020). 31419591 10.1016/j.wneu.2019.08.023

[36] Kabbasch, C. et al. Comparison of WEB embolization and coiling in unruptured intracranial aneurysms: safety and efficacy based on a propensity score analysis. *World Neurosurg*. **126**, e937-e943 (2019). 10.1016/j.wneu.2019.03.016 30862582

[37] Kabbasch, C. et al. WEB embolization versus stent-assisted coiling: comparison of complication rates and angiographic outcomes. *J Neurointerv Surg*. **11.8**, 812-816(2019). 10.1136/neurintsurg-2018-014555 30674636

[38] Pflaeging, M., Kabbasch, C., Schlamann, M., Pennig, L., Juenger, S. T., Grunz, J. P., ... & Goertz, L. (2021). Microsurgical clipping versus advanced endovascular treatment of unruptured middle cerebral artery bifurcation aneurysms after a “coil-first” policy.*World Neurosurgery*,*149*, e336-e344. 10.1016/j.wneu.2021.02.027 33607288

[39] Goertz, L., Liebig, T., Siebert, E., Pennig, L., Laukamp, K. R., Celik, E., ... & Kabbasch, C. (2021). Woven Endobridge embolization versus microsurgical clipping for unruptured anterior circulation aneurysms: a propensity score analysis.*Neurosurgery*, *88* (4), 779-784. 10.1093/neuros/nyaa539 33372215

